# Assessing RNA atomistic force fields via energy landscape explorations in implicit solvent

**DOI:** 10.1007/s12551-024-01202-9

**Published:** 2024-06-17

**Authors:** Konstantin Röder, Samuela Pasquali

**Affiliations:** 1 Randall Centre for Cell & Molecular Biophysics, King’s College London, London, SE1 1UL UK; 2 Laboratoire Biologie Functionnelle Et Adaptative, CNRS UMR 8251, Inserm ERL U1133, Université Paris Cité , 35 Rue Hélène Brion, Paris, France

**Keywords:** RNA, Force fields, Energy landscapes

## Abstract

Predicting the structure and dynamics of RNA molecules still proves challenging because of the relative scarcity of experimental RNA structures on which to train models and the very sensitive nature of RNA towards its environment. In the last decade, several atomistic force fields specifically designed for RNA have been proposed and are commonly used for simulations. However, it is not necessarily clear which force field is the most suitable for a given RNA molecule. In this contribution, we propose the use of the computational energy landscape framework to explore the energy landscape of RNA systems as it can bring complementary information to the more standard approaches of enhanced sampling simulations based on molecular dynamics. We apply the EL framework to the study of a small RNA pseudoknot, the *Aquifex aeolicus* tmRNA pseudoknot PK1, and we compare the results of five different RNA force fields currently available in the AMBER simulation software, in implicit solvent. With this computational approach, we can not only compare the predicted ‘native’ states for the different force fields, but the method enables us to study metastable states as well. As a result, our comparison not only looks at structural features of low energy folded structures, but provides insight into folding pathways and higher energy excited states, opening to the possibility of assessing the validity of force fields also based on kinetics and experiments providing information on metastable and unfolded states.

**Supplementary Information:**

The online version contains supplementary material available at 10.1007/s12551-024-01202-9.

## Introduction

RNA molecules are key not only to the synthesis of proteins based on genomic information but furthermore are involved in a plethora of regulatory processes (Staple and Butcher 2005; Ponting et al. 2009; Siomi et al. 2011; Ken et al. 2023). These functions of RNA molecules are strongly related to their three-dimensional structure (Cruz and Westhof 2009). Complicating the structure function relationship for RNAs is the fact that RNA molecules exhibit polymorphism by which multiple different configurations at comparable energies may be adopted by a given RNA sequence. These competing structures are linked to more complex energy landscapes, where often multiple funnels are observed—a feature associated with multifunctionality (Röder and Wales 2018a). This relationship has been shown explicitly for a range of systems, including RNA tetraloops (Chakraborty et al. 2014) and 7SK RNA (Martinez-Zapien et al. 2017; Röder et al. 2020).

The complexity of possible RNA structures is enriched by so called non-canonical interactions. While Watson–Crick base pairing, which is dominant in DNA, is also observed in RNA stems, it is complimented by a large number of non-canonical nucleobase interactions. Over 150 of such interactions have been identified (Leontis and Westhof 2001; Stombaugh et al. 2009), and they are essential for the stability of structural features such as base triplets. These interactions, alongside the stacking of nucleotides and a wealth of electrostatic interactions, result in a rich and diverse set of three-dimensional structures.

These intertwined features, non-canonical pairing and polyomorphism, lead to fundamental problems for studies of RNAs, both in experiment and computationally. From the multifunnel character of the energy landscape the following behaviour arises: Either multiple structures rapidly interconvert such that individual structures cannot be characterised well, or the structures are separated by high energy barriers, such that they may be characterised, but the transition between them is so slow that it is difficult to identify all relevant structures (Wales and Salamon 2014). The complex interactions of nucleotides require high temporal and spatial resolution in experiment and, for computational work, high accuracy for these intricate arrangements. As a result, it is challenging to resolve RNA structural ensembles, and often multiple methods are used in tandem to study RNAs (Shi et al. 2020; Orlovsky et al. 2020; Alderson and Kay 2021; Röder et al. 2022b).

In this contribution, we propose the study of energy landscapes as a tool to assess RNA force fields. As a first application, we compute the energy landscape of an RNA pseudoknots for different potential energy functions of the AMBER family, commonly used in computational studies of RNA. These are classical force fields based on allatom energy functions including harmonic bonds and angles, sinusoidal functions for torsions, Coulomb interactions for charges and partial charges and a Lennard–Jones potential to account for Van der Waals interactions and excluded volume. The various force fields differ in the parameters used in these commons functional forms. To obtain these force fields, the original force fields developed for proteins and double stranded DNA have been modified to better describe single-stranded RNA molecules. Much effort has been spent on improving the dihedral potentials for RNA (Wang et al. 2000; Pérez et al. 2007; Zgarbova et al. 2011; Yildirim et al. 2010; Aytenfisu et al. 2017; Tan et al. 2018), with some optimisation of van der Waals interactions (Tan et al. 2018) and electrostatics (Steinbrecher et al. 2012). The various force fields have been validated extensively in MD simulations of relatively small systems with the aim to reproducing the correct folds and local behaviour (see (Sponer et al. 2018) for an extensive review). There are fewer results, performed with enhanced sampling MD simulations on small systems such as tetraloops (Tan et al. 2018; Kuhrova et al. 2016; Bottaro et al. 2016), on how well the different force fields reproduce thermodynamic and kinetic properties of RNAs, but we are not aware of such systematic studies for RNAs large enough to adopt multiple structures. As a consequence, there is no general agreement which force field is best suited to study RNA molecules of biologically significant size.

We believe that validation of RNA force fields should take into account not only the prediction of low energy folds and short time scale behaviour, but larger scale rearrangements and partially folded states. The structural polymorphism observed for RNAs means that the definition of a native, correctly folded state, as it is commonly defined for proteins, is not possible (Vicens and Kieft 2022). Instead, computational studies of RNAs must describe the structural ensemble in its entirety. Studying the full structural ensemble may be achieved by considering the energy landscape of a molecular system, which contains all necessary information to calculate thermodynamic, kinetic and structural properties. Additionally, due to the unique topography associated with the complex interactions encountered in biomolecules, the landscape in itself provides an interesting method for characterisation of a molecular system. We propose that explicit explorations of energy landscapes of RNA molecules with different all-atom force fields will provide a unique way of evaluating the RNA force fields.

Here, these explorations are based on the potential energy landscape framework (Joseph et al. 2017; Röder et al. 2019). The framework in principle could be used with explicit and implicit solvent; however, the use of explicit solvent would result into most computational time being spent on sampling the solvent and not the solute. The use of implicit solvent within the EL framework provides a way to sample RNA conformational space well, while accepting some limitations on the accuracy of predictions. A more detailed discussion of these points is provided in the supporting information (see Section S2.1). While this is clearly a limitation on what we can currently predict using the framework, especially for highly charged systems like RNAs, implicit solvent models have proven successful in studying the folding of RNA tetraloops (Nguyen et al. 2015), where a full exploration of the conformational space is now also possible in explicit solvent (Tan et al. 2018; Miner et al. 2016; Kuhrova et al. 2019; Zerze et al. 2021). Studies of the effects of mutations and methylation in larger RNA hairpins also show good agreement with experimental observations and were fully capable of explaining experimental findings (Röder et al. 2020; Röder et al. 2022a). More recently, the first successful prediction of a large RNA structure using an implicit solvent model was obtained by the Perez group as part of the latest RNA puzzle competition (private communications, publication under review). We therefore have reasons to believe that the global features of the energy landscapes are also captured by implicit solvent models, allowing us to compare the overall topography of the energy landscapes. In the supporting material, we provide some further analysis on the impact of different implicit solvent models (Section S1) and the effect of explicit solvation for RNA structures found in this study (Section S2).

It is worth noticing that in this work, we focus on potential energy landscapes only. The reason for this choice is that the potential energy landscape is directly defined by the potential energy function. This allows us to compare the effects of the different potential energy functions (i.e., force fields) on the structural ensemble. Free energy landscapes may be obtained from the potential energy landscape using the harmonic superposition approach (Strodel and Wales 2008), but were not considered here.

In this study, we focused our analysis on the five RNA force fields currently available with the AMBER software. While all force fields are in good agreement with respect to the lowest energy folded structures, there are significantly deviations in the higher energy regions between the force fields. These differences lead to qualitatively different folding path predictions, highlighting the need for careful validation of potentials.

There are other, more recent RNA force fields developed via further analysis of the backbone torsions (Chen et al. 2022; Li et al. 2022), modifications of the hydrogen bond energies either guided by the knowledge of the native structure (Kuhrova et al. 2016) or more in general as additional term in the force field to minimally perturb other interactions (Kuhrova et al. 2019). In the future, an extension of our work to these force fields will be desirable.

## Methodology

### The example system

The choice of RNA for this study combined several prerequisite. The RNA must be small enough to enable good sampling within the energy landscape framework. At the same time, it must be large enough to show distinct structural features, both in the low and higher energy states. Finally, experimental reference points beyond structure are required, especially for the higher energy behaviour. Our choice of RNA, fulfilling these criteria, is the Pseudoknot1 (PK1) from the thermophilic bacterium *A. aeolicus*.

It is the smallest predicted transfer-messenger RNA with 21 nucleotides. Pseudoknots are a common RNA motif, which is not sequence dependent, and challenges RNA structure prediction (Lescoute et al. 2005; Kucharík et al. 2016; Antczak et al. 2018). It shows structural complexity, while computational costs are reasonable due to its size. Last but not least, pseudoknots are functionally highly relevant (Staple and Butcher 2005), for example, in rybozyme catalysis (Ke et al. 2004), regulation of gene expression (Peselis and Serganov 2014) and frameshifting (Shen and Tinoco 1995; Michiels et al. 2001; Nixon et al. 2002). PK1 specifically is required for the ribosomal rescuing mechanism of trans-translation found in bacteria (Nameki et al. 2000).

**Fig. 1:**
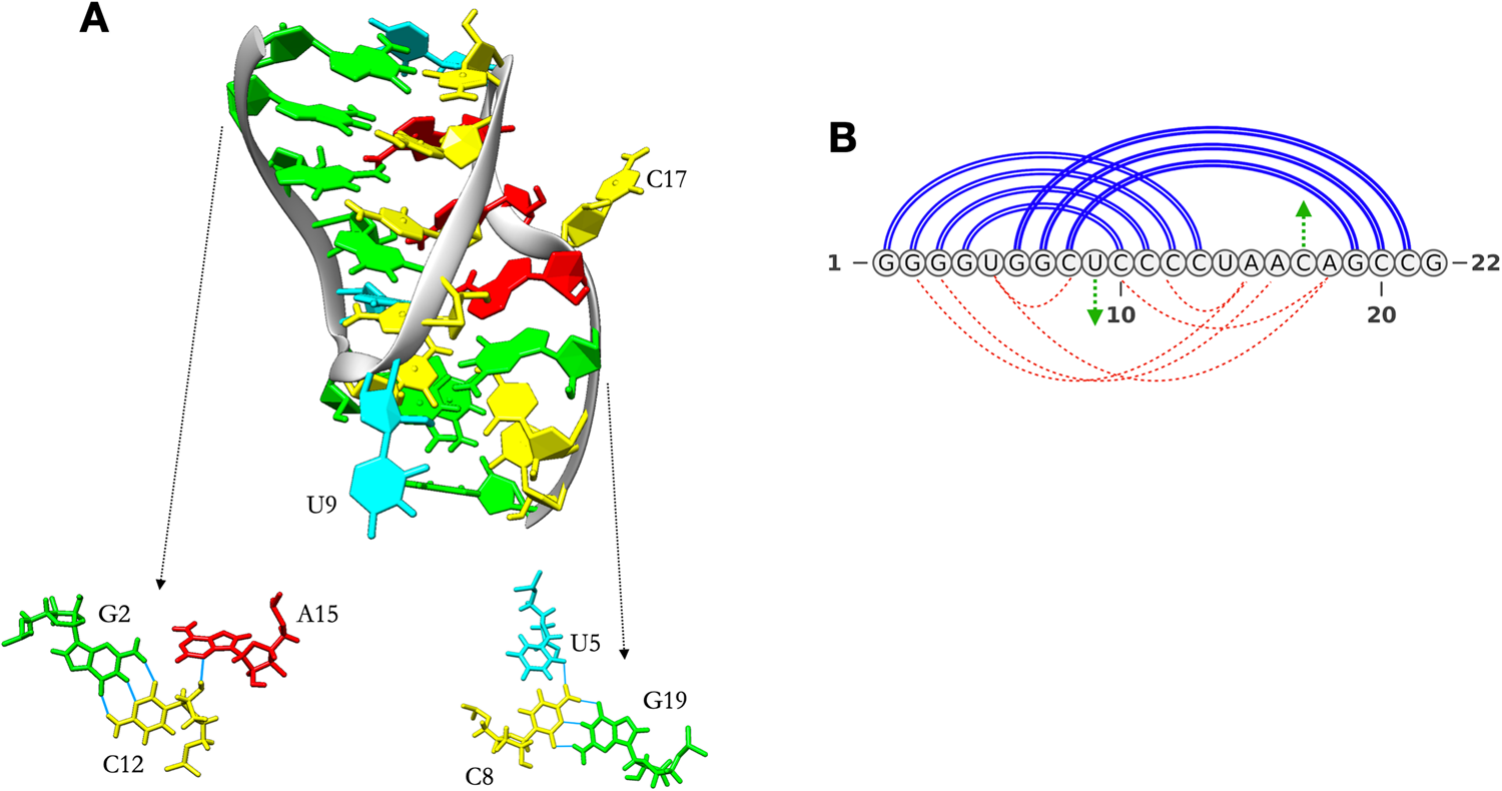
Key features of the PK1 structure. **A** Three-dimensional structure of PK1 (PDB entry: 2G1W) and details of two non-canonical interactions as an example. Nucleotides are colour coded according to the NDB standard (red for A, green for G, yellow for C and cyan for U). **B** Arc diagram of the secondary structure of PK1, highlighting the two stems formed (blue lines), the non-canonical pairings (red dashed lines) and the bases pointing toward the exterior of the structure (green arrows)

The structure of the PK1 pseudoknot, resolved by NMR with 14 proposed configurations (Nonin-Lecomte et al. 2006), is an H-type pseudoknot characterized by two stems of four and three canonical base pairs, respectively (stem 1, G1–G4 paired with C10–C13; stem 2, G6-C8 paired with G19–C21). The RNA forms a tight fold with the two stems aligning almost on top of each other (Fig. 1). One base in each loop (U9 and C17) points out of the structure, while the other bases in the loops form an extensive network of non-canonical interactions with the stems: U5 forms non-canonical interactions with C8 and A18, A15 with G2 and C12, A16 with G3 and finally A18 with U5 and C10 (Fig. 1 A and B). The system exhibits a particularly high melting temperature (56 °C in 50 mM NaCl and 73 °C with 1 M MgCl_2_ added), which the authors of the NMR study attribute to the tight network of interactions.

### Force field specifications

**Fig. 2:**
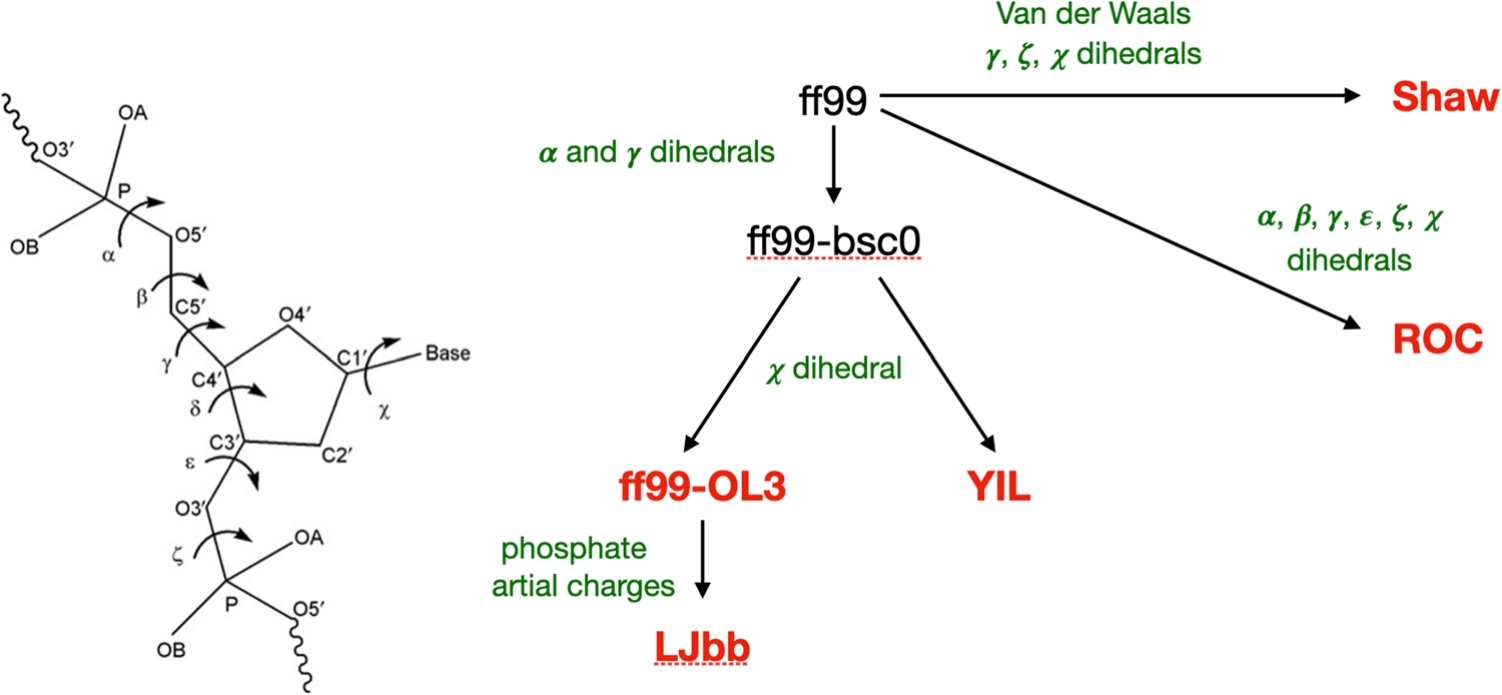
Scheme of the modifications introduced by the five force fields analysed in this study (in red), starting from the common AMBER force field ff99

The starting point of the most currently used AMBER RNA force fields is the ff99 force field, derived through modification of sugar pucker and glycosidic torsion (*χ*) (Wang et al. 2000) compared to older force fields. Then, ff99-bsc0 was derived through modification of the *α* and *γ* dihedral by the group of M. Orozco by fitting high-level quantum mechanical calculations (Pérez et al. 2007). A new modification of *χ* from more accurate quantum chemical calculations also accounting for possible solvation errors by Jurenčka, Sponer, Otyepka and co-workers led to ff99-OL3 (Zgarbova et al. 2011; Banáš et al. 2010), which is now the AMBER recommended force field for single-stranded RNA simulations. A separate modification of *χ* by the Turner group based on NMR data let to the YIL force field (Yildirim et al. 2010). A further optimization of ff99-OL3 electrostatic interactions (partial charges) by the group of D. Case led to LJbb (Steinbrecher et al. 2012). More comprehensive reparameterizations were conducted by the group of Shaw on van der Waals parameters and dihedrals from quantum chemical calculations and experimental data to derive the Shaw force field (Tan et al. 2018). The Rochester research group of Mathews derived the ROC force field from quantum chemistry calculations optimization. The relation between this force field variants is sketched in Fig. 2.

The energy landscape framework uses geometry optimisation to locate transition states (TS), from which in turn we can find the two minima connected by a given TS. If explicit solvation is used, this would require sampling of all solvent transition states and local minima (or at least a representative sample thereof). Effectively, this would lead to sampling solvent configurations for a given solute state (see SI for more detail).

Therefore, our current efforts use implicit solvent, but we actively work at devising solutions to bring water and ions back into the picture.

In this work, we compare the energy landscapes of PK1 for these five force fields ff99-OL3, YIL, LJbb, Shaw and ROC with an implicit solvent description through a Generalised Born approximation (*igb* = 2). Because each force field is parameterised including water and ions, and that not all models use the same water models, the use of implicit solvent may impact the comparison of different force fields, and therefore care is needed in drawing detailed conclusions on the performance of each model. For example, the LJbb model was parameterised specifically to better account for phosphate interactions, and therefore we expect the lack of ions to affect it differently than the other models. Similar reasoning holds for force fields using different water models such as OL3 and Shaw.

In order to probe the impact of the choice of implicit solvent model, we compared different implicit solvent models. For each of the energy landscapes obtained, we drew a representative sample of minima at random and compared their order (lowest to highest energy) with different implicit solvent models. These models include different methods to compute effective radii and alternative implicit solvent models, for example, GB with a surface area contribution. We also compared how the energy ordering would change if we keep the solvent model and change the force field. For more details on the exact procedure, see the supporting material Section S1. We do not find any significant reordering of structures based on the implicit solvent model choice (Figs. S1 to S4), but we do observe reordering, particularly of partially folded structures when the force field is changed (see Fig.S4). These observations not only provide some evidence that the nature of the implicit solvent model is not impacting energy landscape topography, but that the different energy landscape topographies observed are inherent force field features and not sampling artefacts.

### Exploration of the energy landscapes

The discrete pathsampling (Wales 2002, 2004) as part of the computational energy landscape framework (Joseph et al. 2017; Röder et al. 2019) is used to obtain kinetic transition networks (Noé and Fischer 2008; Wales 2010). Low energy minima for the system were located with basin-hopping global optimisation (Li and Scheraga 1987, 1988; Wales and Doye 1997) initiated from unfolded structures and NMR structures from PDB entry 2G1W (Nonin-Lecomte et al. 2006). These minima were used to seed the energy landscape for OL3. We then used a fully folded, a partially folded and an unfolded structure from this landscape to seed the energy landscape exploration for the other four force fields. This approach assumes that none of the force fields will produce erroneous very low-energy structures with significantly different folds compared to the experimentally observed pseudoknot and lower computational costs. Transition states were located with the doubly nudged elastic band algorithm (Henkelman and Jónsson, H. 2000; Henkelman et al. 2000; Trygubenko and Wales 2004) with quasi-continuous interpolations (Carr and Wales 2005; Röder and Wales 2018b). The candidates for transition states were converged with hybrid eigenvector following (Munro and Wales 1999).

Convergence of the EL was assessed in two ways. Firstly, some algorithms used to find new transition states converge automatically, as they sample around specific features such as kinetic traps or high barriers. Secondly, the overall convergence was assessed by whether the appearance (topography) and thermodynamic properties change when new minima are added.

Disconnectivity graphs (Becker and Karplus 1997; Wales et al. 1998) are used to represent the energy landscapes and aid the identification of funnels, which contain distinct conformations. For the key basins, we extract all RNA structures and analyse their average properties. This procedure, as shown previously (Röder et al. 2022a), enables us to describe features of distinct RNA structures including the dynamic variations within each set.

For each ensemble of structures, we monitor average properties of each nucleotide by looking at values of dihedral angles and puckering and at the number of stacking and base paring interactions formed. In this work, we used the Barnaba software (Bottaro et al. 2019) to extract these properties for each structure in the ensemble. The secondary structures represented here are those of the lowest energy minimum in each basin. We have computed the dot-bracket representation for all structures of each ensemble and verified that they are consistent with only minimal fluctuations for each funnel.

## Results and discussion

**Fig. 3:**
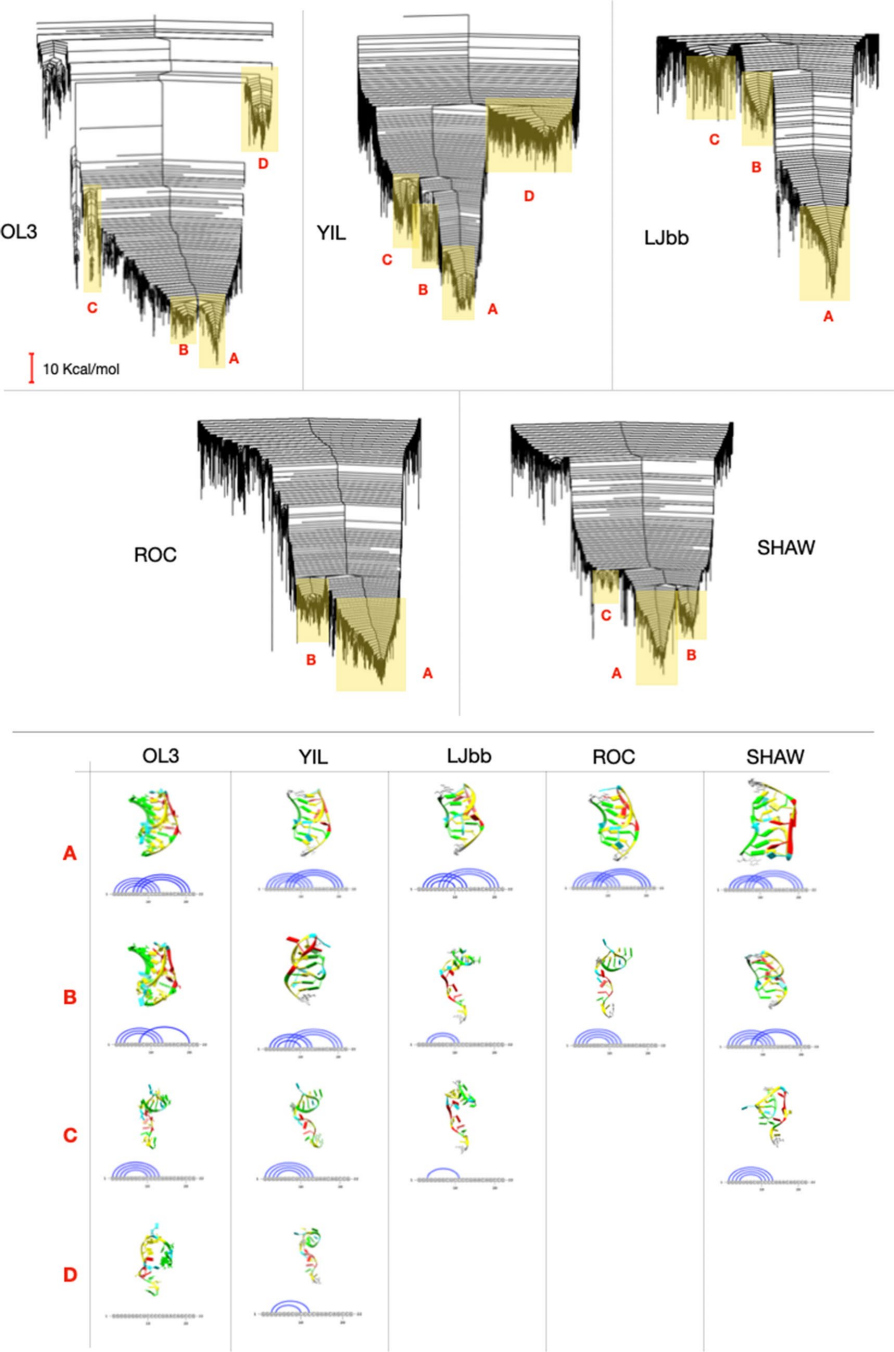
Top: The potential energy landscape of PK1 obtained with the five different force fields. For each potential, the disconnectivity graph is shown, and basins of interest for further analysis are highlighted in yellow. Each branch (vertical line) corresponds to a local minimum. Branches merge at the energy when there is a transition path between them that is entirely lower in energy. This analysis is conducted in discrete steps, chosen here as 1 kcal/mol. All graphs are on the same scale. The ordering of minima on the horizontal axis is based on how branches split of the parent node and avoids crossing of branches. In this way, the graphs faithfully represent the organisation of the energy landscape into different funnels. Bottom: Two- and three-dimensional structure of the lowest energy minimum for each basin highlighted on the energy landscapes of each model

The energy landscapes for the five force fields are illustrated in Fig. 3 at the same scale. It is noticeable that the landscapes differ in their appearance, with different numbers of funnels and variation in the energy difference between funnels. The assembly of pseudoknots depends on the relative stability of its helical segment (Cho et al. 2009), and it is therefore expected that folding in this pseudoknot is initiated by folding of the longer 5^′^ helical segment. Since the stem with 4 base pairs (stem 1) is more stable than the one with only 3 (stem 2), partially folded states with stem 1 formed are to be expected. Indeed, such folding has been reported for other H-type pseudoknots as well (Staple and Butcher 2005; Shen and Tinoco 1995). While we see such states, their stability with respect to the unfolded and the fully folded pseudoknot varies significantly across the force fields.

### Structure of the fully formed pseudoknot

**Fig. 4:**
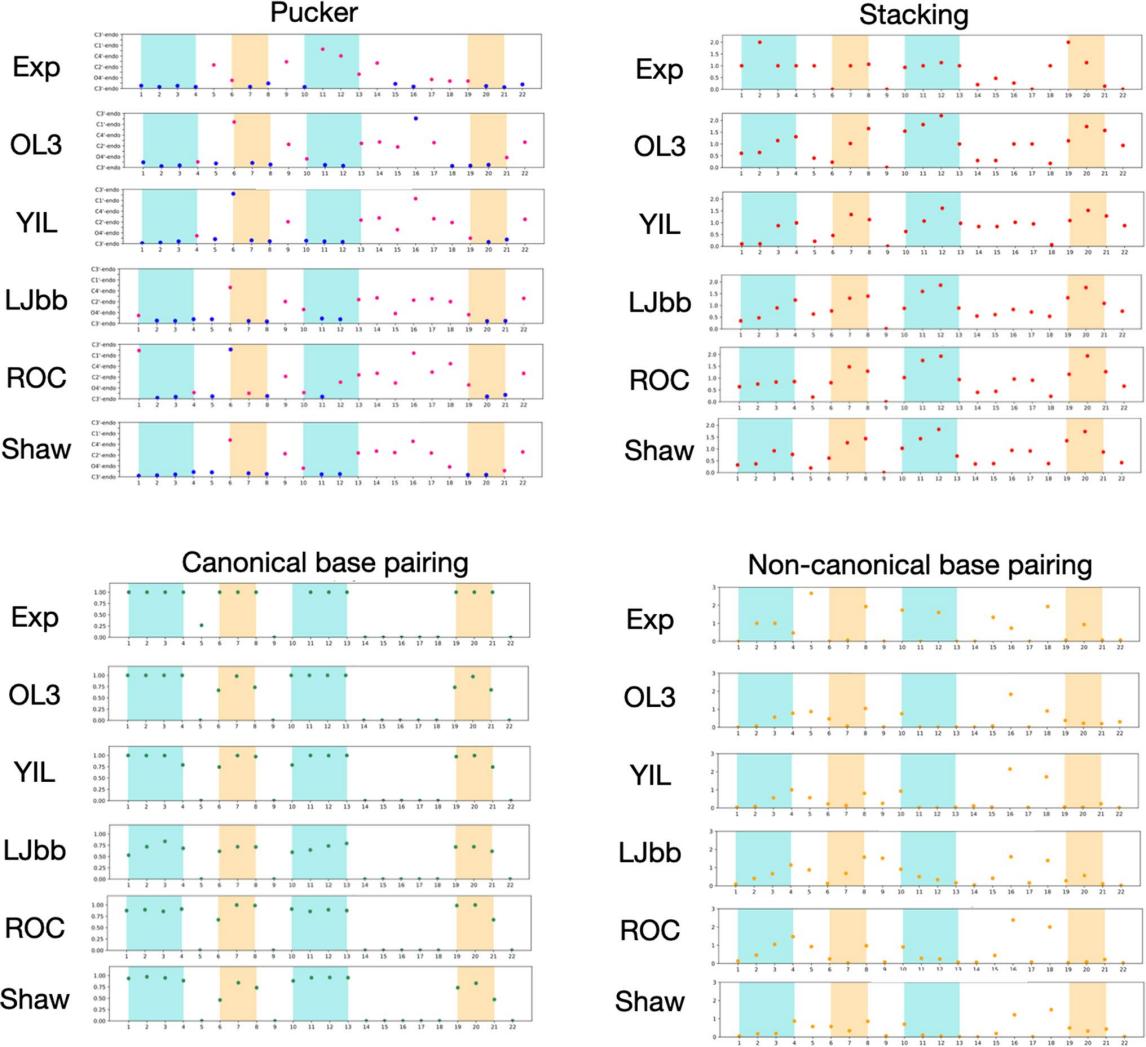
Average puckering, stacking interactions, canonical and non-canonical base pairing for each nucleotide in the sequence for the global minimum (basin A in all models) for the five force fields and for the experimental NMR structures. Shaded areas correspond to stem 1 (cyan) and stem 2 (beige) of the native structure and are reported as reference

The first part of our analysis centres around the accuracy of the pseudoknot structure found with the different force fields as compared to the experimental structure. Figure 4 reports the structural features for each nucleotide for the global minimum basin for the five force fields. The reported features are the averages of all structures in the funnels labelled A in Fig. 3.

The puckering varies slightly compared to the experimental structure, especially for residues 5, 11 and 12 (stem 1). This is a significant observation as both residues 5 and 12 are involved in multiple base pairs in the native structure. This link is reflected by the non-canonical base pairs formed by these two bases, which is reduced compared to the experimental structures. Overall, there is a stronger tendency to stack in all force fields, with higher nucleotide stacks than in the experimental structure. Excessive stacking can be a consequence of the implicit solvent; however, the overstabilisation of stacking is also well known for fully solvated models (Morgado et al. 2009; Banáš et al. 2012). Improved force field parameters can alleviate these issues to some extent (Bergonzo and Cheatham 2015).

The five models share further common structural features apart from the presence of the two stems. All models correctly predict U9 pointing to the outside of the structure and not making any stacking or hydrogen bonds. They also predict C17 not making any canonical or non-canonical interactions, but they all predict some staking for it. All models predict the triplet A18-G4-C10 and G2-C12-A15 or its variant with G3 in place of G2. In contrast, the non-canonical interactions involving C8, C10 and C12 are mostly not present. The strong stacking interactions change the position of the nucleotides such that they cannot form the non-canonical interactions observed in experiment. For the YIL force field, only one triplet is formed. For LJbb, the base paring in stem 1 is incomplete and similar to a higher energy metastable state for the YIL force field, lacking base paring for C11. As for non-canonical interactions, there are two triplets, A18-G4-C10, corresponding to the NMR structure, and G3-C12-A15, which is a shift from G2 to G3 with respect to experimental structure.

The fact that we obtain the native structure as the most stable configuration for all models, even in the absence of ions, which for pseudoknots can be critical, is encouraging in the assumption that even with the implicit solvent the force fields obtain sensible structures. This observation is further supported by the fact that reported structures are stable in short explicit solvent MD simulations (see SI Section S2).

### Energy landscape topography and partially folded states

For the OL3 force field, which is the currently recommended force field by the AMBER developer, we observe three distinct sets of structures: the folded pseudoknot (basin A), partially folded states (basins B and C, where stem 2 (the 3^′^ stem) has disappeared) and unfolded states (D). These states are clearly separated in energy and enable the qualitatively correct folding sequence based on helical stability.

The YIL force field, which differs from OL3 in the glycosidic dihedral angle, *χ*, shows a deep, main funnel. At the bottom of this funnel, we find, as expected, the folded pseudoknot structures. There are two smaller, higher-energy subfunnels with significant energy barriers to the folded pseudoknot. The lower one of the two (B) is a pseudoknot, but lacking the base pairing for C11. No experimental evidence suggests that these structures exist. The higher subfunnel (C) contains structures with stem 1 formed. Finally, a shallow basin with some residual base pairing in stem 1 exists at high energy (D).

LJbb is based on OL3 with changed electrostatics. The energy landscape exhibits the most pronounced funnel for the pseudoknot structure. Metastable structures with only stem 1 formed are high in energy compared to the folded pseudoknot. With this energy landscape organisation, collective rather than step wise folding is likely, a qualitatively different folding behaviour compared to for example OL3. As described above, the global minimum has a different base pairing than the experimental structures. When analysing the entire set of structures in the basin (A), the base pairs missing are formed in around 50% of cases. This result shows that the shifted and the correct structures are both present, but without any significant barriers between them. Assessing the structures in the funnel for their secondary structure, the state corresponding to experimental base pairs lies about 1 kcal/mol above the global minimum.

The energy landscape obtained from ROC exhibits a large, deep funnel whose global minimum (A) is the native state. A second smaller funnel (B), higher up in energy, corresponds to structures with only stem 1 formed. Puckering is rather different from the experimental values and also from the previously discussed models, as expected since the previous three were all derived from ff99-bscO. Differences are particularly significant for residues holding a key role in the overall structure such as U5 and the nucleotides of the second loop, A15 to, A16, C17 and A18 that are responsible for many non-canonical interaction in the experimental structure. As the previous models, the network of non-canonical interaction is not as extensive as in the experimental structure, with fewer interactions for U5 and for the bases already involved in stems; however, the triplets A18-G4-C10 and G2-C12-A15 are correctly formed.

The Shaw force fields generate a landscape with a funnel exhibiting several metastable states. The global minimum corresponds to the native structure. A second pronounced funnel higher in energy exhibits and alternative fold with stem 2 correctly formed but stem 1 formed by only 3 base pairs with an off-shift of one base. Even higher in energy, we find a small funnel with the structures with only stem 1 formed. The global minimum (A) lacks one of the base pairs of stem 2, but the correct fold is found at and energy of 0.9 kcal/mol higher and belongs to the same funnel. Once more, the network of non-canonical interactions is less extended than in the experimental structure, but both triplets A18-G4-C10 and G2-C12-A15 are correctly formed. Stacking in the stems is more pronounced than for the native structure, and pucker also differs significantly for U5 and nucleotides 15, 16 and 17.

Summarising the differences in the topographies of the energy landscapes, the following picture emerges. OL3 and ROC have only one clearly identifiable metastable partially folded state corresponding to structures with only stem 1 formed, LJbb does not have any partially folded states, while YIL and Shaw have basins with structures alternative to native for stem 1 and at higher energies have partially folded states with stem 1 formed. OL3, YIL and LJbb present small funnels at high energies of structures stabilized by stacking and by one or two base pairs, while the presence of such funnels is less clear in ROC and Shaw. No model exhibits basins with only stem 2 formed, in agreements with thermodynamic expectations.

The energies necessary for crossing barriers between basins vary significantly. The transition form native structure (A) to the lowest energy partially folded structure (B) for OL3 is around 20 kcal/mol and of 90 kcal/mol from native to fully unfolded (D).

ROC’s transition from native to partially folded (B) is higher, with an energy of about 40 kcal/mol. The transition from native (A) to the alternative fold (B) in YIL is of 50 kcal/mol with the transition to the partially folded structure (C) of 60 kcal/mol and to the almost fully unfolded (D) of 80 kcal/mol. This last value is similar to that of LJbb with a barrier from native to almost fully unfolded (B) of 90 kcal/mol. Shaw’s barrier between native fold (A) and alternative fold (B) is of 30 kcal/mol with a transition to the partially folded state (C) at 40 kcal/mol. Overall, OL3 has the lowest transition energy between fully folded and partially folded state, ROC and Shaw have comparable transition energies between native folds and partially folded structures, slightly higher than those of OL3, while YIL and LJbb have deeper native basins with transition energies some 20–40 kcal/mol higher for the partially unfolded state with respect to OL3 and comparable energies for the transition from folded to fully unfolded.

## Conclusion

In this contribution, we examined the performance of five currently available atomistic force fields for RNA simulations from AMBER. Our study analyses not only the ability of a force field to predict correctly folded structures, in this case a H-type pseudoknot, but furthermore, by using energy landscape explorations, the metastable states and their relative energies.

Our work mainly wants to show the potential of the energy landscape analysis in assessing the performance of different force fields, testing their behaviour in regions of the conformational space distant from those commonly used for their optimization, near the native states. This is particularly relevant when looking at the full behaviour of biological molecules for which one is interested in thermodynamics and kinetics that do depend on the presence of metastable states higher in energy.

Despite the use of implicit solvent, which is a significant limitation of our exploration, we believe that we can still draw some general conclusion on the performance of the five force fields we analysed. For the pseudoknot structure, we observe broad agreement between the force fields and reasonable accuracy compared to known NMR structures. A key difference between experimental and simulated structures is the overstabilisation of stacking interactions over non-canonical interactions, although this might in part be based on the implicit solvation required for energy landscape explorations. It should be noted, however, that the stacking directly prevents certain non-canonical interactions from forming, and we do not observe these interactions at higher energy either. This observation hints at the fact that non-canonical interactions are still not fully represented in the current set of force fields, in agreement with tetraloop simulations in fully solvated systems that revealed an underestimate of hydrogen bonding energies and that were at the origin of the HBfix (Kuhrova et al. 2016) and gHBfix (Kuhrova et al. 2019). For two bsc0 derived force fields, namely LJbb and YIL, we further identify alternative structures with incomplete stem base pairing, which are in fact lower in energy than the expected fully formed pseudoknot.

When considering the overall topography of the energy landscape and the partially folded, metastable states, more significant differences between force fields emerge. A good point of comparison is the expected folding pathway, which can be predicted based on thermodynamic considerations and observations in similar pseudoknots. As the two stems are of different lengths, it is expected that stem 1, the 5^′^-stem, forms first, before the second stem is folded for the full pseudoknot. Indeed, this process is clearly seen in the OL3 force field and the Shaw and Rochester modifications—although even these three force fields differ in the energy differences between the partially folded and the fully folded pseudoknot structures. In contrast, the LJbb modification to OL3 destabilises the partial folds so much that a cooperative rather than step-wise folding mechanism would be predicted. To a lesser extent, this is also observed for the Yildrim modifications. The fact that two of the five force fields predict potentially qualitatively different folding paths clearly highlights the need for (a) a better understanding of the behaviour of force fields far from the native states, (b) wider parametrizations schemes including non-native states and (c) broader criteria for testing RNA force fields.

For RNA, structural polymorphism is a key observation, and as a result, computational methods must be able to represent competing folded and partially folded structures to capture the dynamical heterogeneity of RNA structure. While more established methods based on molecular dynamics simulations have difficulties in fully exploring the competition between alternative states, the exploration of the energy landscape that we propose is able to address this question extensively. The main approximation of our approach is in the use of implicit solvent, especially critical for a system so sensitive to the environment and to the presence of ions such as RNA. However, in this framework, we are able to systematically consider systems of significant size, much larger and complex in architecture than what previously done in fully solvated systems. We observe large differences in the energy landscapes, that are both qualitative, with the presence of different metastable states, and quantitative, with a wide range of energy barriers to be crossed between similar states, and that can hardly be attributed to the lack of solvent only. We therefore believe that our work, despite approximations that any method intrinsically carries along, constitutes an informative complement to the detailed analysis performed on smaller systems. Well aware of the importance of solvent and ions for RNA systems, we actively work at extending our energy landscape framework to account for solvent, possibly combining our current exploration of the landscape with explicit solvent molecular dynamics simulations.

While this is an intense conceptual and computational effort, we hope to be able to repeat the comparison of the energy landscapes in explicit solvent in a not too far. While the performance benchmark on large conformational transitions is not yet achieved, the ability of several force fields to capture qualitatively correct ordering of reasonable structures is promising. In our opinion, this result shows that qualitative work on RNA structural transitions is feasible with the current generation of force fields and a broad range of complementary sampling techniques.

### Supplementary Information

Below is the link to the electronic supplementary material.

Supplementary file1 (PDF 2717 KB)

## Data Availability

The energy landscape databases are available on zenodo, 10.5281/zenodo.10590336.
