# Supplementary material for: Assessing RNA atomistic force fields via energy landscape explorations in implicit solvent
Source: Biophys Rev. 2024 Jun 17;16(3):285–95. doi: 10.1007/s12551-024-01202-9 (PMC11297004; doi:10.1007/s12551-024-01202-9)
Supplement: Supplementary file 1 — Supplementary file1 (PDF 2717 KB) [file 12551_2024_1202_MOESM1_ESM.pdf]

# Assessing RNA atomistic force fields via energy landscape explorations in implicit solvent – Supplementary Material

Konstantin Röder[1*] and Samuela Pasquali[2*]

[1*]Randall Centre for Cell & Molecular Biophysics, King's College London, London SE1 1UL, United Kingdom.
[2]Laboratoire Biologie Functionnelle et Adaptative, CNRS UMR 8251, Inserm ERL U1133, Université Paris Cité, 35 rue Hélène Brion, Paris, France.

*Corresponding author(s). E-mail(s): konstantin.roeder@kcl.ac.uk ; samuela.pasquali@u-paris.fr;

## S1 Effects of different implicit solvent models

Within the AMBER simulation suite, there are a number of different Implicit solvation models available. The most commonly used implicit solvent model is the Generalised Born (GB) model, which was used in this study. The motivation for using implicit solvation lies in the reduced computational cost, as a) fewer interactions have to be calculated and b) sampling is restricted to solute rather than solvent and solute configurations. Several options exist to estimate the effective radii within the GB scheme.

Two sets of these have been shown to provide reasonable agreement with explicit solvation simulations and the Poisson-Boltzmann equation, namely OBC models (igb=2 and 5 [1, 2]) and the GBn model (igb=7 [3] and 8 [4]).[a] In addition to these models, an additional term for the surface area can be included into the calculation. The two options for this extensions considered here are surface area computation with the LCPO model (GBSA1, [5]) and using a fast pairwise approximation (GBSA3, [6]). An alternative implicit solvent model that can be used is the Analytical Linearized Poisson-Boltzmann (ALPB) approximation [7, 8]. ALPB tends to be more accurate than the GB model, but has been less extensively tested. Within this approximation, the igb models are used to obtain effective radii.

## S1.1 Approach

Resampling the entire energy landscape with all possible implicit solvent models is unfeasible due to computational costs, as there are 16 different options within the described models (GB, GBSA1, GBSA3 and ALPB with igb 2, 5, 7 or 8). We used the available databases for the five force fields (LJbb, OL3, ROC, Shaw and YIL) that have been sampled using GB with igb2. We then selected 40 minima from each database. In order to have a reasonable spread between low and high energy minima, we randomly drew 4 minima from each energy decile. For the highest energy decile, we discarded minima at very high energies (more than 100 kcal/mol above the lower decile boundary). We then recalculated the energy for each of these minima with the 16 different solvation model combinations.

In a second set of calculations we used only the GB igb2 and GBSA1 igb2 options and reoptimised every minima from one force field with the other four force fields by minimising the energy. Optimisations ran for a maximum of 2500 steps.

Both sets were analysed by plotting the energies of the minima as function of the models using a rainbow colour scheme to distinguish each decile.

[a] The igb options for the OBC and GBn correspond to different parameterisations within the models.

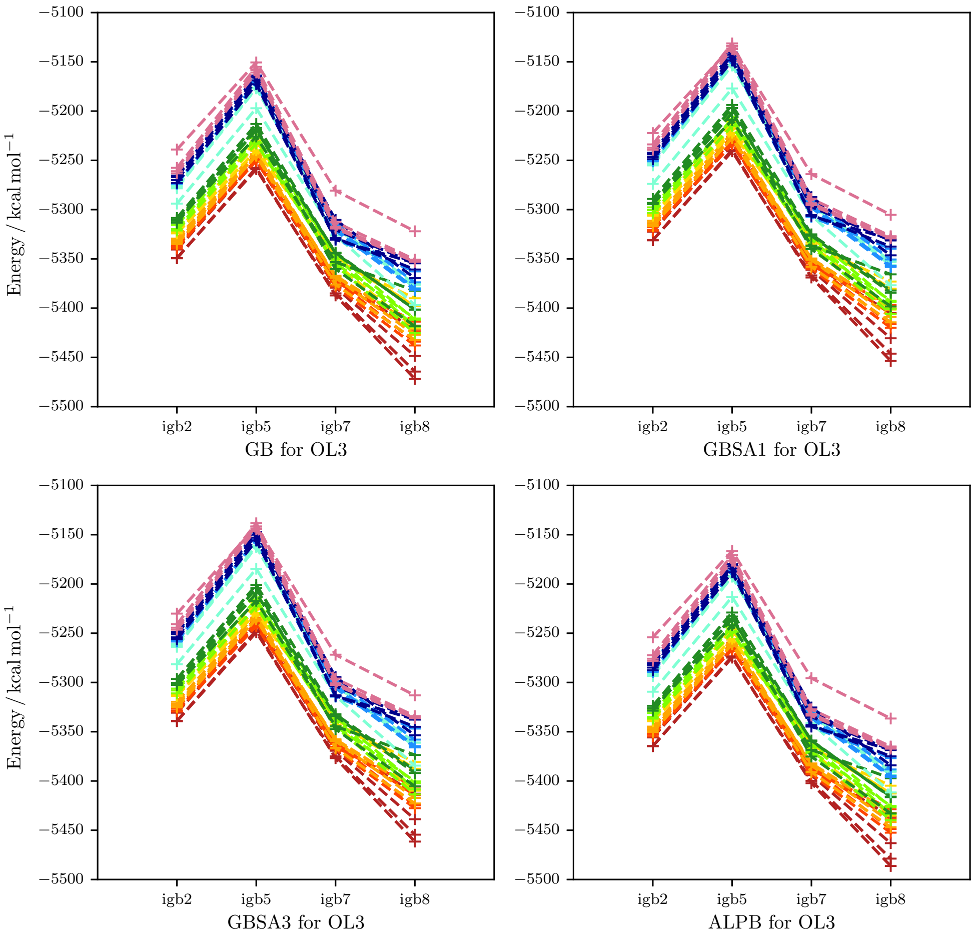


**Fig. S1** Comparison of different effective radii computations for the GB, GBSA1, GBSA3 and ALPB models for minima from the OL3 energy landscape. While the absolute energies shift between the different radii computations, we do not notice a significant reordering of the minima.

## S1.2 Results

**Effective radii.** In Fig. S1, the change in energy of the minima from the OL3 energy landscape due to changes in the method of computing effective radii (i.e. the chosen igb model) is shown for the different implicit solvation methods. The overall trends between solvation methods are similar, apart from changes in the absolute energy (see below). The energy for the different radii computations differ significantly with the

lowest energy minimum varying from around -5500 kcal/mol for ALPB and igb8 to -5250 kcal/mol for GBSA models with igb5. However, these shifts are conserved across all methods, and the shifts also broadly conserve the energy ordering of the minima. We observe a small number of changes between deciles for igb7 and igb8 compared to igb2 and 5, i.e. between GBn and OPC models.

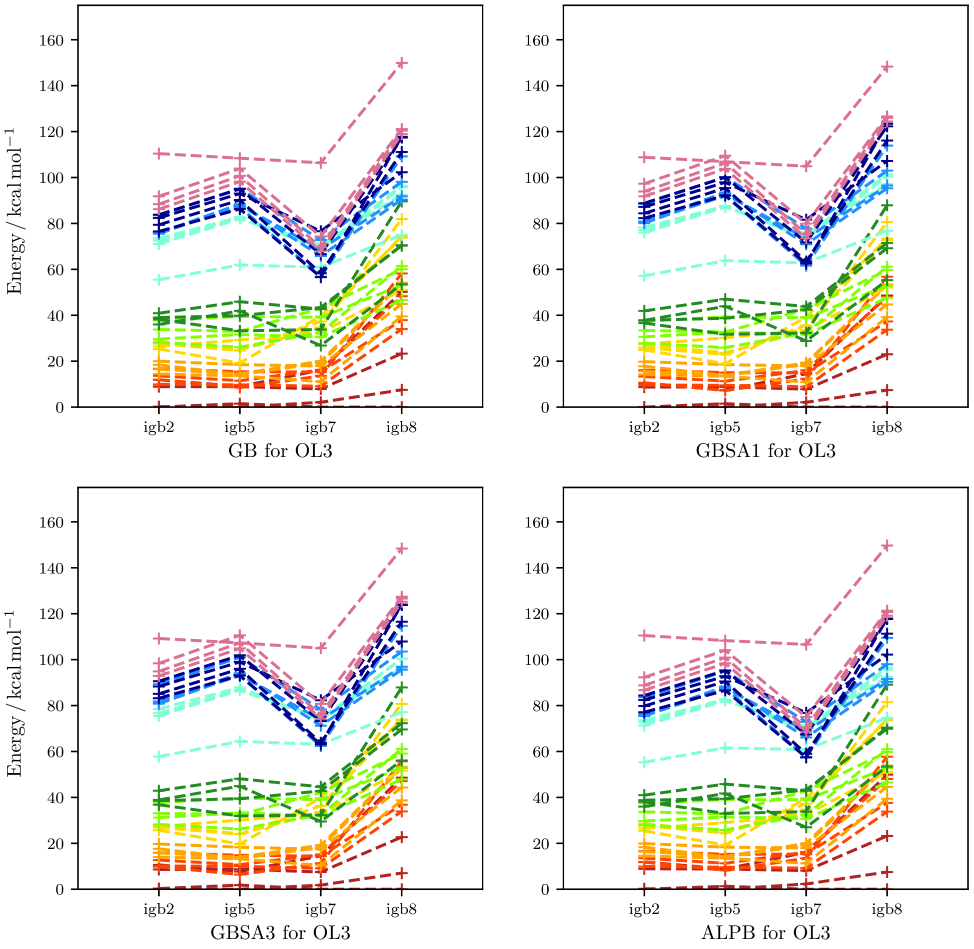


**Fig. S2** Comparison of different effective radii computations for the GB, GBSA1, GBSA3 and ALPB models for minima from the OL3 energy landscape as in Fig. S1, but setting the lowest energy minimum to 0.

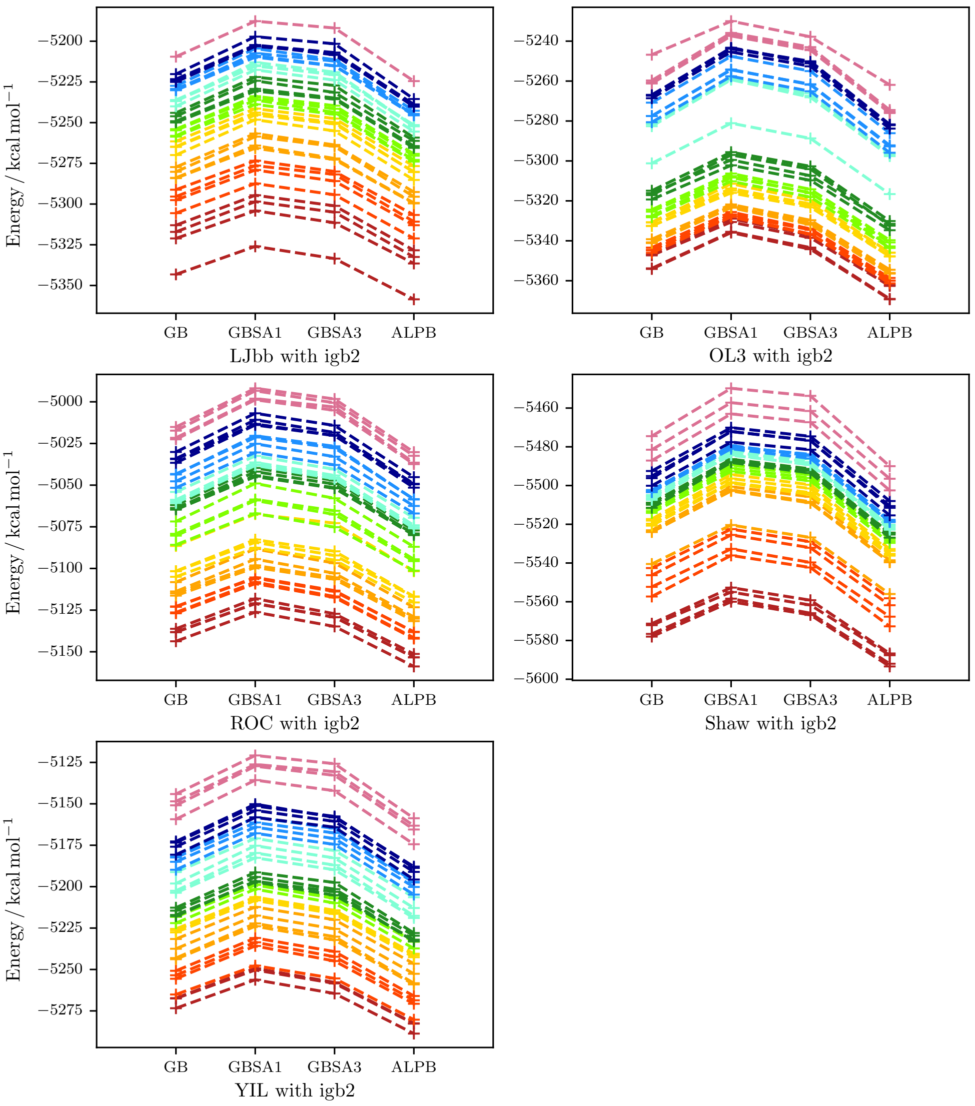


**Fig. S3** Comparison of GB, GBSA and ALPB for all five force fields using effective radii from igb2. Consistent trends are observed across all force fields without reordering of energies.

Fig. S2 shows the same data as Fig. S1, but we shift all energies uniformly for each set of energies, such that the energy of the lowest energy minimum is zero. This representation enables a better view of the shift in energy scale between the igb models and the reordering between models. The most reordering occurs for igb7, while the

other models are far more consistent. Perhaps this observation is not surprising as the model is not "recommended for systems involving nucleic acids" (AMBER Manual 2023).

**Implicit solvent approximations.** While Fig. S1 already indicates that there only small differences between the models, Fig. S3 shows the comparison between different methodologies (GB, GBSA and ALPB) for the different force fields using igb2. While the absolute energies change between models and there are minor changes in the energy differences between minima, the overall energy order is conserved. We observe no changes in energy that would impact the structure of the energy landscape, if the surface area is included or ALPB is used compared to the GB model.

**Alternative force fields** Fig. S4 shows changes to the energy order of minima for each force field when another one of force field is used instead. In this data set, we see significantly more reordering than for the different implicit solvation models. While the lowe energy minima are broadly preserved in their order, the medium and high energy deciles are significantly shuffled, showing the impact of force field changes. This finding is in line with the overall observation of distinct energy landscape topographies in this study.

## S1.3 Summary

We find small, non-significant changes between implicit solvent models for the samples of structures taken form the energy landscapes. Overall, the variation between force fields is much more isgnificant than the impact of the implicit solvation model. In addition, we recover this behaviour when we study the changes in the order of minima upon reminimisation with a different force field. While the solvation model choice will impact calculated observables, we do not find any evidence that these changes will impact the energy landscape topography.

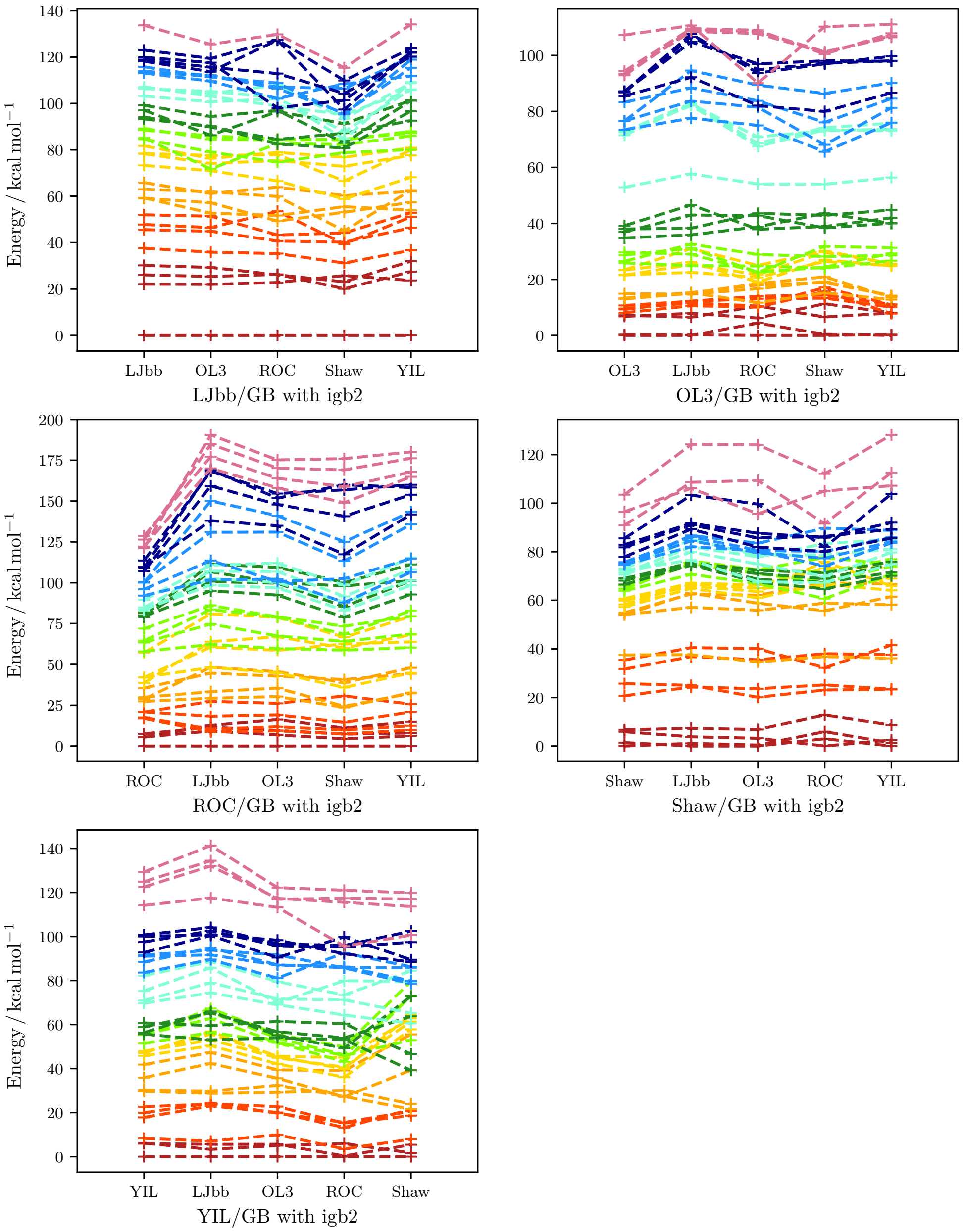


**Fig. S4** Changes to the energy order of minma if a different force field is used. The first set in each plot is the force field used for the landscape that the minima are taken from. The other four sets in each plot are the other four force fields. The deciles are colour-coded revealing the changes in ordering of the minima. The energy of the lowest energy minimum was set to zero for each set to illustrate changes in energy differences and ordering better.

# S2 Effects of explicit solvation

Beyond the different implicit solvation models, explicit solvent can be used in molecular simulations. Again, there are many different models available, and some models are particularly suitable for a given force field.

## S2.1 Explicit solvent in the EL framework

A key challenge in using explicit water in simulations is convergence. While explicitly accounting for water and ions increases the accuracy of simulations, the simulations require not only sampling of the solute but also of the solvent. As a result, we are often faced with a trade-off between accuracy and sampling coverage. The energy landscape approach is designed to sample well, but at the moment we are limited to implicit solvent. The reason for this lies in the use of geometry optimisation. Within the framework, we are locating transition states, defined as Hessian index 1 saddle points, and the local minima they connect. Sampling these triples of minimum-TS-minimum then allows us to build a graph representation of the energy landscape. If we were to use explicit solvent, we would need to identify all related transition states between solvent configurations. This would mean our sampling would not just focus on the solute, but mostly on the solvent. A possible way around this challenge lies in new schemes to keep the focal point on the solute, but using explicit solvation. How exactly such a scheme might work is not clear at this stage. As a result, only preliminary data exists to identify the impact of using the implicit solvent. Exploratory work on a small example have found that the topography of the energy landscape is barely impacted by switching to explicit solvation (Private Communications, Dr D. Chakrabarty and Prof. D. J. Wales), but this has not been shown for larger systems.

Some previous work is providing evidence that the framework reproduces observables well despite the use of implicit solvent. This set of research includes comparisons

to other computational studies [9], matching the experimental free energies of structural transitions for a range of point mutations (albeit for a protein) [10], and matching the results from the EL framework with Hamiltonian-replica exchange simulations for an RNA example [11].

Another way to verify the results from energy landscape explorations is to use short explicit solvent MD simulations to verify the stability of predicted structures. We would not expect that the local minima change significantly over a short period of time. This stability should be decreased somewhat at higher energies, i.e. for unfolded or partially folded structures, where we expect faster changes. While we cannot extend such analysis easily to the entire landscape and all minima, it allows us to probe distinct structures that are proxies for the overall structural ensemble.

## S2.2 Approach

For each database, we select between two and four minima, either within the same funnel but different structures, or in different funnels. For each minimum, we add explicit solvent and ions (adjusted based on box size to 0.15M). We used TIP3P for LJbb and ROC, OPC for OL3 and YIL, and TIP4P-D for Shaw. After an initial minimisation with restraints on the solute, we minimised the full system. We then heated the system to 300 K again using constraints on the solute, which we then slowly removed. After a pressure equilibration, we ran 100 ns production runs. For each minimum, we obtained five repeat trajectories, meaning a total of 15 sets of five 100 ns trajectories.

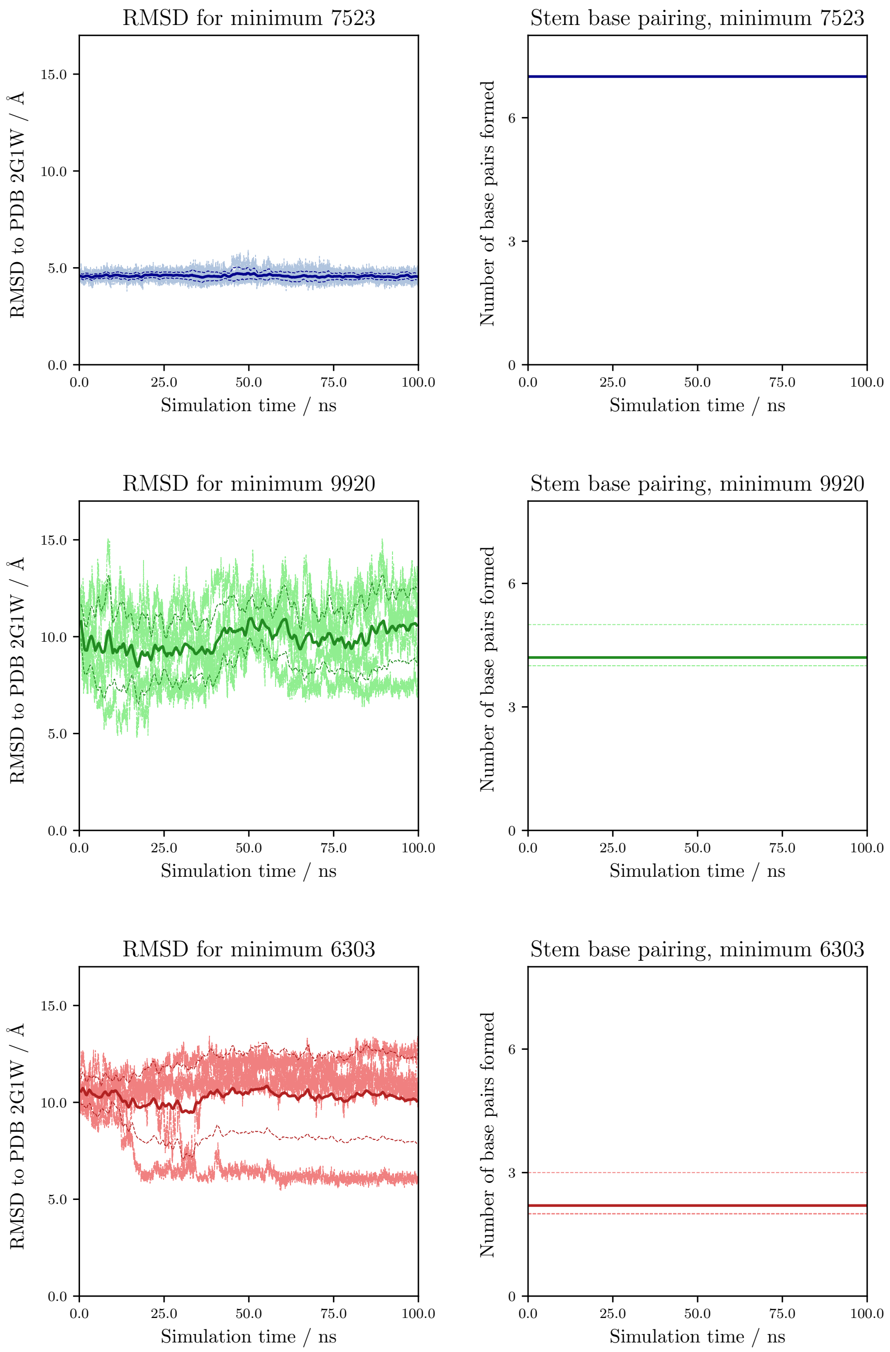


**Fig. S5** RMSD and base pairing in the MD simulations for minima taken from the LJbb energy landscape. The darker solid lines indicate the smoothed average (smoothing window 1 ns) over all repeats with the darker dashed lines providing the standard deviation.

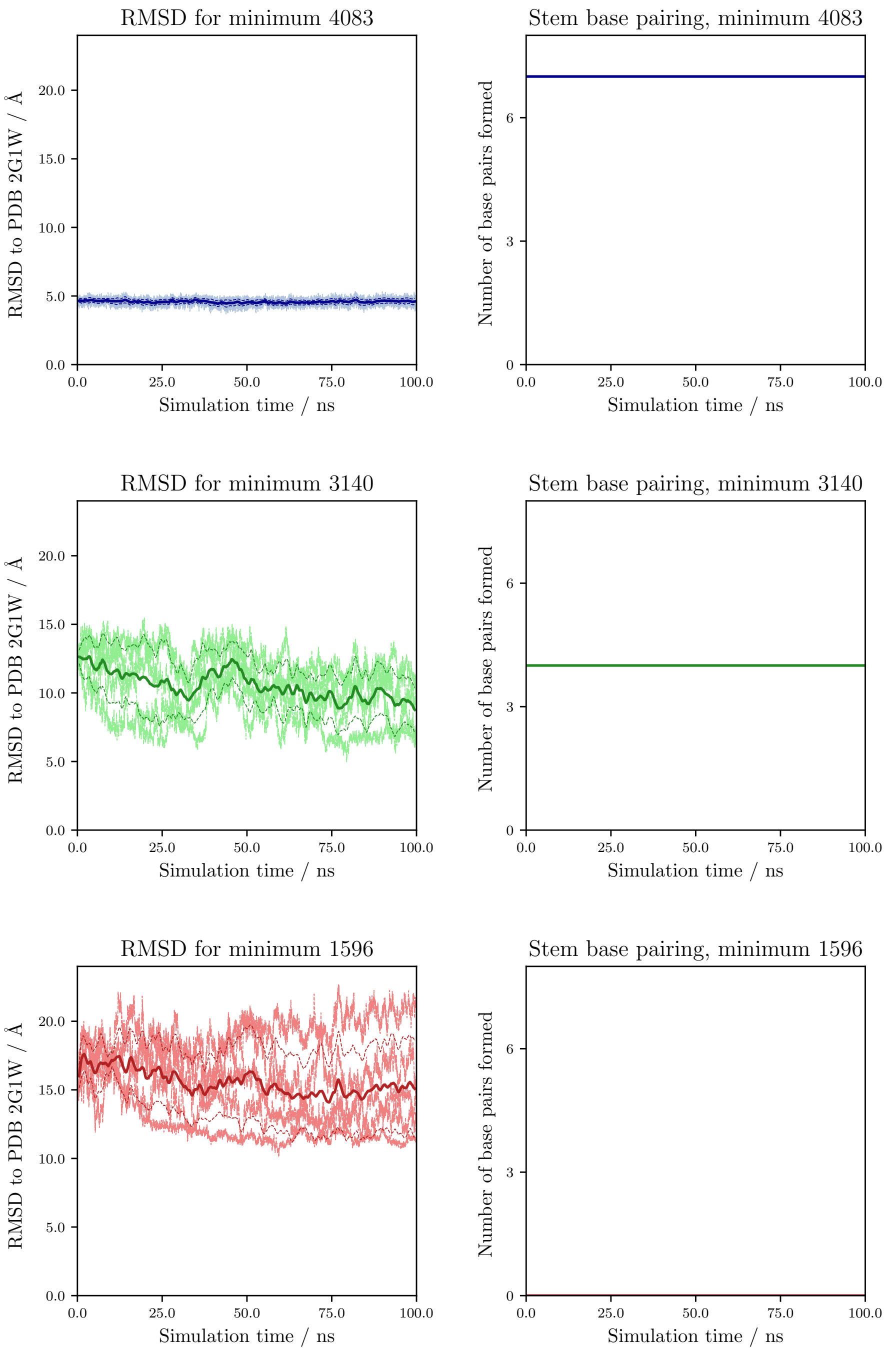


**Fig. S6** RMSD and base pairing in the MD simulations for minima taken from the OL3 energy landscape. The darker solid lines indicate the smoothed average (smoothing window 1 ns) over all repeats with the darker dashed lines providing the standard deviation.

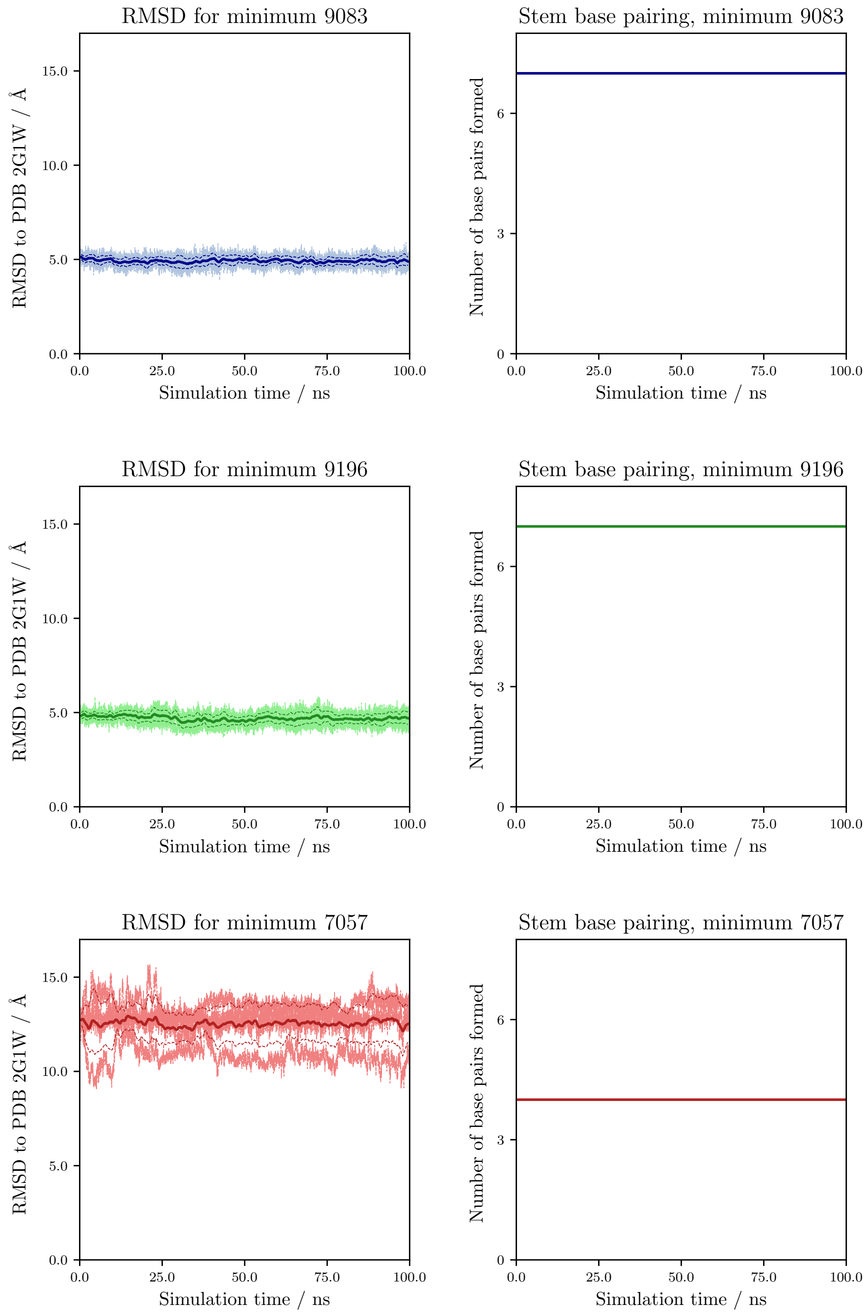


**Fig. S7** RMSD and base pairing in the MD simulations for minima taken from the ROC energy landscape. The darker solid lines indicate the smoothed average (smoothing window 1 ns) over all repeats with the darker dashed lines providing the standard deviation.

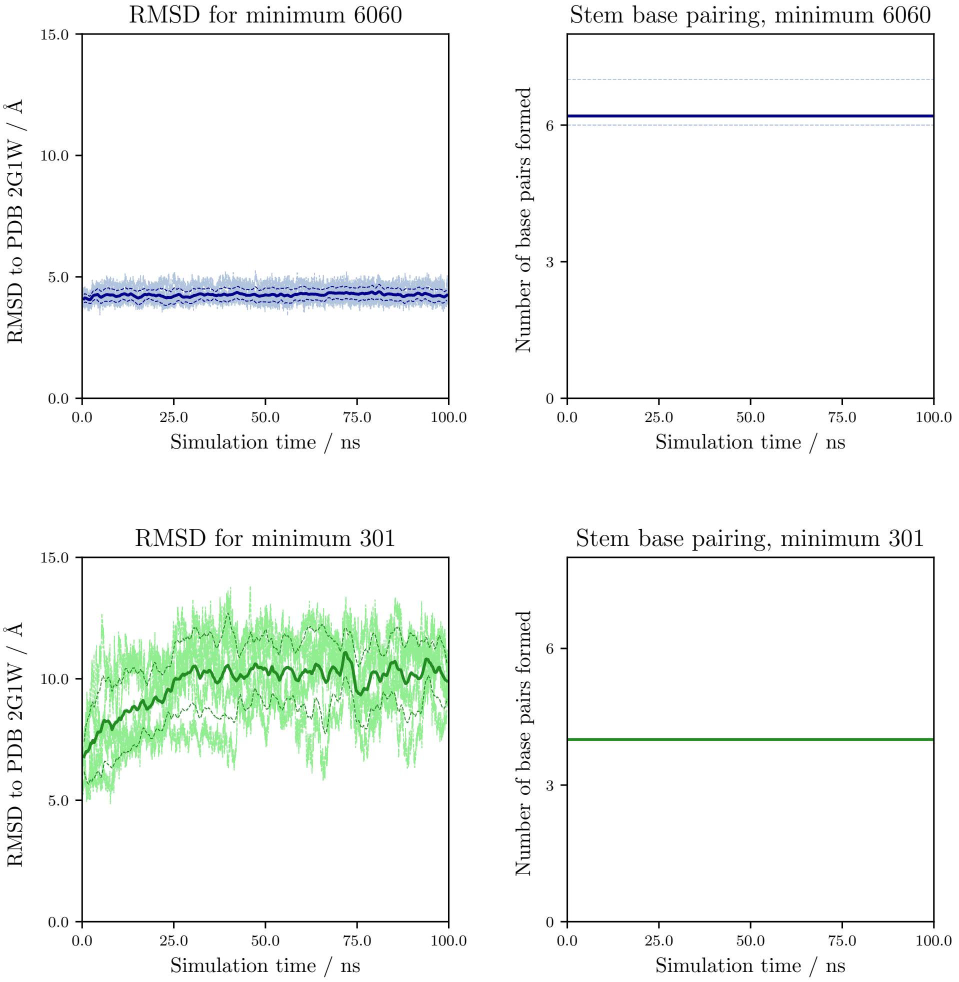


**Fig. S8** RMSD and base pairing in the MD simulations for minima taken from the Shaw energy landscape. The darker solid lines indicate the smoothed average (smoothing window 1 ns) over all repeats with the darker dashed lines providing the standard deviation.

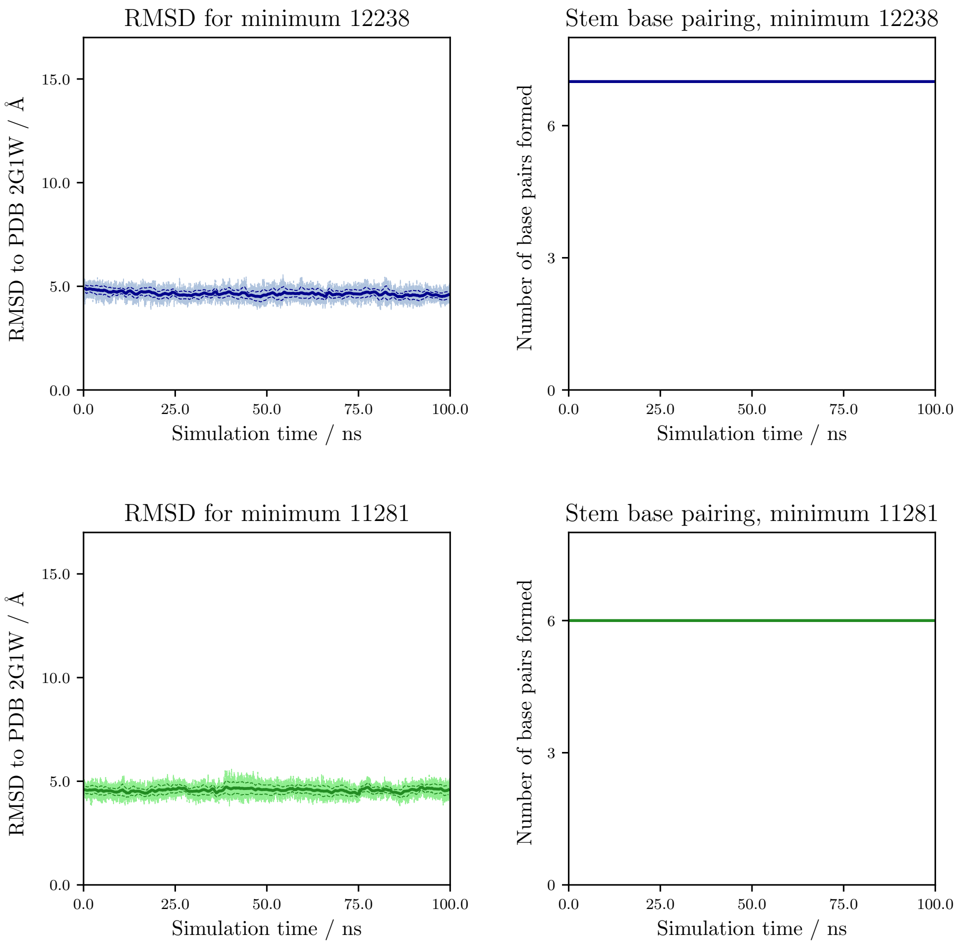


**Fig. S9** RMSD and base pairing in the MD simulations for the first set of minima taken from the YIL energy landscape. The darker solid lines indicate the smoothed average (smoothing window 1 ns) over all repeats with the darker dashed lines providing the standard deviation.

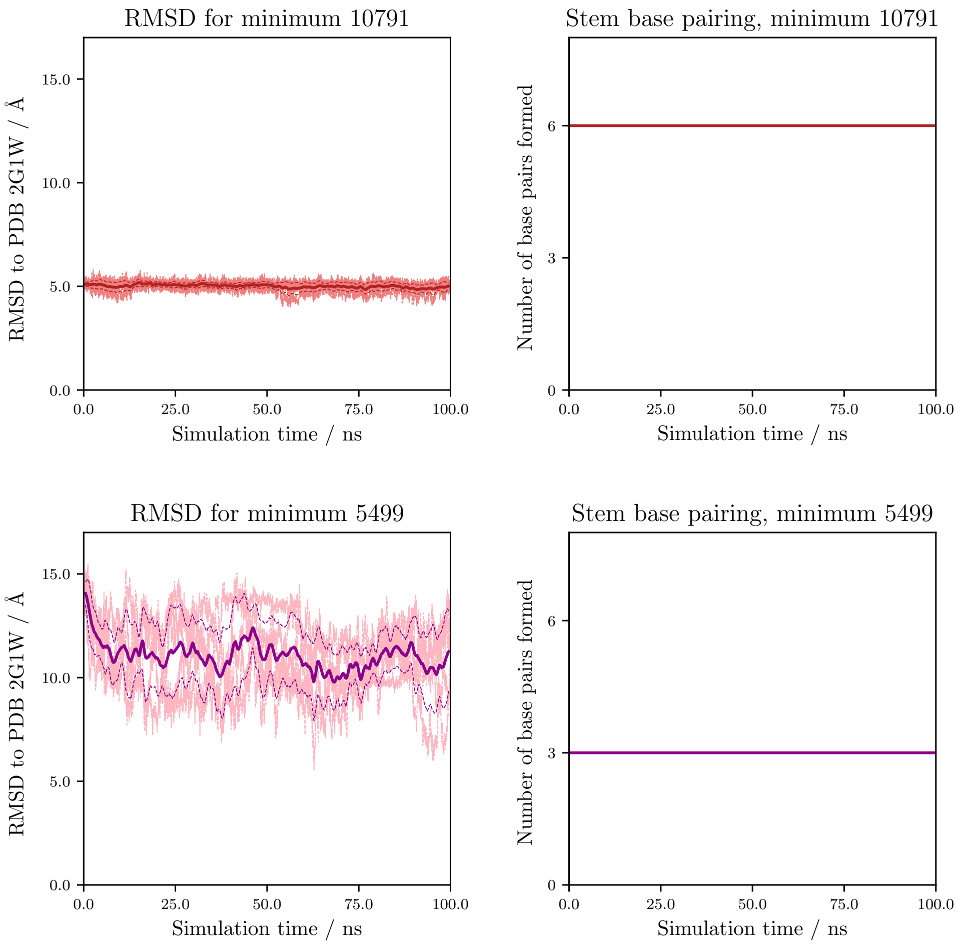


**Fig. S10** RMSD and base pairing in the MD simulations for the second set of minima taken from the YIL energy landscape. The darker solid lines indicate the smoothed average (smoothing window 1 ns) over all repeats with the darker dashed lines providing the standard deviation.

## S2.3 Results

The first analysis of the MD simulations focuses on structural features. Fig. S5 to S10[b] show for each of the force fields two features for each minimum , namely the RMSD to the experimental 2G1W structure and the base pairs found in the two stems. The minima in each figure are shown from the lowest energy minimum at the top to the highest energy minimum at the bottom. For both descriptors, the individual trajectories for all five repeats are shown in a lighter colour, with a smoothed average (smoothing window of 1 ns) shown in a darker colour.

| Force field | Structures from | Minimum 1 | Minimum 2 | Minimum 3 | Minimum 4 |
|---|---|---|---|---|---|
| LJbb | Energy landscape<br>Average from MD | 6.0<br>4.6±0.1 | 15.2<br>9.9±0.6 | 12.9<br>10.3±0.3 | |
| OL3 | Energy landscape<br>Average from MD | 6.1<br>4.6±0.1 | 20.1<br>10.5±1.0 | 18.2<br>15.5±0.9 | |
| ROC | Energy landscape<br>Average from MD | 5.9<br>4.9±0.1 | 5.9<br>4.7±0.1 | 14.5<br>12.5±0.2 | |
| Shaw | Energy landscape<br>Average from MD | 5.2<br>4.3±0.1 | 5.9<br>9.8±0.9 | | |
| YIL | Energy landscape<br>Average from MD | 5.5<br>4.6±0.1 | 7.2<br>4.6±0.1 | 5.2<br>5.0±0.1 | 20.1<br>11.0±0.7 |

**Table 1** Comparison of the RMSD from the experimental PK1 structure 2G1W averaged across the five trajectories with same RMSD for the energy landscape minimum. All values are in Å.

**RMSD.** The RMSD measurement serves two purposes. Firstly, it measures the stability of structures. As detailed above, the expectation is that the structures found are stable for short MD simulations (here 100 ns each). This is observed for the MD simulations, as seen in Fig. S5 to S10. More variation is seen for higher energy structures for each force field, but the average over multiple repeats indicates relative

[b]The figure for the YIL minima is split into two to fit on the page.

stability. The second purpose is to measure changes relative to the minima on the landscape. For this we take a single average over all trajectories for a given minimum. The resulting values and their counter parts for the minima from the energy landscape are provided in Table 1. Broadly, the average RMSD is slightly lower than in the energy landscape picture. This comparison, though, is flawed, as the MD structures, especially for higher energy minim does fluctuate, and a better comparison would be to map the space the MD simulations have explored back to a region of the energy landscape and compare it to an average across all structures in that region. It is not clear, however, how such a mapping could be obtained, as MD trajectories are not sampling minima and transition states only.

**Base pairing.** Perhaps a better measure for the extent of structural changes in this context is therefore the base pairing observed in minima. As described in the main manuscript, we observe the pseudoknot (7 base pairs across the stems), some related, misfolded structures (6 or 7 base pairs), partially folded structures (4 base pairs or one stem formed correctly) and various unfolded or nearly unfolded structures (no or at maximum two base pairs). When considering these indicators of structure, we observe a remarkable conservation of base pairing, even in unfolded/misfolded structures. A prime example is minimum 1596 for the OL3 landscape (bottom of Fig. S6), which is completely unfolded and without base pair, and no base pair is formed in any of the MD trajectories.

This observation shows a) that these structures are not artefacts of implicit solvent use, but features of the force fields, and b) that the structural variation in the RMSD is likely a consequence of motions of unpaired nucleotides and not of base pairs forming or breaking. The overall topography of the landscape is mainly guided by the base pairing within structures. These findings point towards a conservation of the topography reported in explicit solvent.

| Force field | Minimum | Funnel | $E_{\mathrm{EL}}$ | $E_{\mathrm{MD}}$ | $\Delta$ | Structure |
|---|---|---|---|---|---|---|
| LJbb | 7523 | A | 0.00 | 0.00 | 0.00 | Full pseudoknot |
| | 9920 | A | 36.05 | 39.85 | 3.80 | Only 4-bp stem formed[1] |
| | 6303 | A | 62.97 | 65.86 | 2.89 | Partly unfolded 4-bp stem[1] |
| OL3 | 4083 | A | 0.00 | 0.00 | 0.00 | Full pseudoknot |
| | 3140 | C | 29.23 | 48.77 | 19.54 | Only 4-bp stem formed |
| | 1596 | D | 75.34 | 90.25 | 14.91 | Fully unfolded |
| ROC | 9083 | A | 0.00 | 0.00 | 0.00 | Full pseudoknot[2] |
| | 9196 | A | 5.03 | -6.82 | -11.85 | Full pseudoknot[2] |
| | 7057 | B | 17.45 | 25.50 | 8.05 | Only 4-bp stem formed |
| Shaw | 6060 | B | 0.00 | 0.00 | 0.00 | Misfolded pseudoknot |
| | 301 | C | 16.46 | 17.36 | 0.90 | Only 4-bp stem formed |
| YIL | 12238 | A | 0.00 | 0.00 | 0.00 | Full pseudoknot |
| | 11281 | A | 8.89 | -4.56 | -13.45 | Misfolded pseudoknot |
| | 10791 | B | 17.27 | 9.58 | -7.70 | Misfolded pseudoknot |
| | 5499 | C | 27.66 | 34.41 | 6.75 | Partial folded stemloop |

**Table 2** Comparison of the energy of the minima used in explicit MD simulations from the energy landscape explorations, $E_{\mathrm{EL}}$, and the average MD energy at 5 ns steps across the five repeats for each minimum, $E_{\mathrm{MD}}$. Energies are relative to the lowest energy minimum for each force field. The difference between the energy differences, $\Delta$, is provided as measure of changes in ordering of the structures.

## S2.4 Energy comparisons

A final point of comparison between MD simulations and EL explorations is the energy of the individual structures. A direct comparison is difficult as the solvation changes the energy function between MD and EL exploration. Furthermore, the solvation boxes for different minima are not identical, meaning we have a different number of molecules for each minimum. As a rather crude proxy we take the configuration of each minimum for each trajectory at 5 ns steps. We then remove solvent and ions and recalculate the energy with the original function used in the EL explorations. We then get an average energy for each minimum, enabling us to calculate energy differences between structures. This process yields an energy ordering, and the results are shown in Table 2.

In general, the energy gaps increase during the MD simulations, especially between partially folded or unfolded minima and the canonical pseudoknot. The only exceptions

are alternative folds observed for ROC and YIL, that become more stable than the reference structures. This increased stability is not from altered base pairing (see above). The exact nature of the changes leading to the reordering in energy is not clear, but given the approach and the fact that the only structures changing order are in the same funnel, there is again no evidence that the explicit solvent would alter the topography of the energy landscape.

# References


[1] Onufriev, A., Bashford, D., Case, D.A.: Modification of the generalized born model suitable for macromolecules. J. Phys. Chem. B **104**, 3712–3720 (2000)

[2] Onufriev, A., Bashford, D., Case, D.A.: Exploring protein native states and large-scale conformational changes with a modified generalized born model. Proteins **55**, 383–394 (2004)

[3] Mongan, J., Simmerling, C., McCammon, J.A., Case, D.A., Onufriev, A.: Generalized born with a simple, robust molecular volume correction. J. Chem. Theory Comput. **3**, 156–169 (2007)

[4] Nguyen, H., Roe, D.R., Simmerling, C.: Improved generalized born solvent model parameters for protein simulations **9**, 2020–2034 (2013)

[5] Weiser, J., Shenkin, P.S., Still, W.C.: Approximate atomic surfaces from linear combinations of pairwise overlaps (lcpo). J. Comput. Chem. **20**, 217–230 (1999)

[6] Huang, H., Simmerling, C.: Fast pairwise approximation of solvent accessible surface area for implicit solvent simulations of proteins on cpus and gpus. J. Chem. Theory Comput. **14**, 5797–5814 (2018)

[7] Sigalov, G., Scheffel, P., Onufriev, A.: Incorporating variable environments into

the generalized born model. J. Chem. Phys. **122**, 094511 (2005)

[8] Sigalov, G., Fenley, A., Onufriev, A.: Analytical electrostatics for biomolecules: Beyond the generalized born approximation. J. Chem. Phys. **124**, 124902 (2006)

[9] Šponer, J., Bussi, G., Krepl, M., Banáš, P., Bottaro, S., Cunha, R.A., Gil-Ley, A., Pinamonti, G., Poblete, S., Jurečka, P., Walter, N.G., Otyepka, M.: RNA structural dynamics as captured by molecular simulations: A comprehensive overview. Chem. Rev. **118**(8), 4177–4338 (2018)

[10] Röder, K., Wales, D.J.: Analysis of the ub to ub-cr transition in ubiquitin. Biochemistry, (2018)

[11] Röder, K., Stirnemann, G., Dock-Bregeon, A.-C., Wales, D.J., Pasquali, S.: Structural transitions in the rna 7sk 5′ hairpin and their effect on hexim binding. Nucleic Acids Research **48**(1), 373–389 (2020)
